# The Effects of Exercise Training on Mitochondrial Function in Cardiovascular Diseases: A Systematic Review and Meta-Analysis

**DOI:** 10.3390/ijms232012559

**Published:** 2022-10-19

**Authors:** Ai Yin Lim, Yi-Ching Chen, Chih-Chin Hsu, Tieh-Cheng Fu, Jong-Shyan Wang

**Affiliations:** 1 Healthy Aging Research Center, Graduate Institute of Rehabilitation Science, Chang Gung University, Taoyuan 333, Taiwan; 2 Department of Information Management, Chang Gung University, Taoyuan 333, Taiwan; 3 Heart Failure Center, Department of Physical Medicine and Rehabilitation, Chang Gung Memorial Hospital, Keelung 204, Taiwan; 4 Research Center for Chinese Herbal Medicine, College of Human Ecology, Chang Gung University of Science and Technology, Taoyuan 333, Taiwan

**Keywords:** exercise, cardiovascular system, oxidative capacity, mitochondrial function

## Abstract

Mitochondria dysfunction is implicated in the pathogenesis of cardiovascular diseases (CVD). Exercise training is potentially an effective non-pharmacological strategy to restore mitochondrial health in CVD. However, how exercise modifies mitochondrial functionality is inconclusive. We conducted a systematic review using the PubMed; Scopus and Web of Science databases to investigate the effect of exercise training on mitochondrial function in CVD patients. Search terms included “mitochondria”, “exercise”, “aerobic capacity”, and “cardiovascular disease” in varied combination. The search yielded 821 records for abstract screening, of which 20 articles met the inclusion criteria. We summarized the effect of exercise training on mitochondrial morphology, biogenesis, dynamics, oxidative capacity, antioxidant capacity, and quality. Amongst these parameters, only oxidative capacity was suitable for a meta-analysis, which demonstrated a significant effect size of exercise in improving mitochondrial oxidative capacity in CVD patients (SMD = 4.78; CI = 2.99 to 6.57; *p* < 0.01), but with high heterogeneity among the studies (I^2^ = 75%, *p* = 0.003). Notably, aerobic exercise enhanced succinate-involved oxidative phosphorylation. The majority of the results suggested that exercise improves morphology and biogenesis, whereas findings on dynamic, antioxidant capacity, and quality, were inadequate or inconclusive. A further randomized controlled trial is clearly required to explain how exercise modifies the pathway of mitochondrial quantity and quality in CVD patients.

## 1. Introduction

Cardiovascular disease (CVD) is a health crisis with increasing incidence and prevalence, which may compromise physical performance and contribute to mortality, morbidity, and the economic burden of health care. The prevalence of CVD remains high and is persistently the leading cause of death globally as data shown estimated 17.9 million people died from CVDs in 2019, contributing to 32% of all global deaths [1]. Mitochondria dysfunction is greatly implicated to the pathogenesis and progression of CVD, setting out the emergence of mitochondrial-targeting therapy [2]. As a non-pharmacological tool, exercise training is potentially a safe and effective measure to restore mitochondrial health in CVD [3]. Nevertheless, the mechanisms of exercise on mitochondrial function are not yet elucidated.

Optimal mitochondrial function is fundamentally achieved through the balance of organelle synthesis and degradation in response to energy demand. Under stable conditions, mitochondrial turnover is regulated by the fusion and fission processes. Any damage to the mitochondrial network will recruit fission protein to cleave off the dysfunctional site. The damaged mitochondrial portion will then be cleared by mitophagic degradation, while the functional portion will be fused to other mitochondria. In a high energy-demand state, such as exercise or under hypoxia condition, the rate of mitochondrial biogenesis and turnover is enhanced. Healthy mitochondria consume oxygen and synthesis ATP in the electric transport chain with an efficient degradation of the damaged site. However, disruption in mitochondrial turnover were seen in many CVD, namely atherosclerosis, reperfusion injury, and cardiomyopathy [4].

Mitochondrial dysfunction manifested with structural and functional abnormality in the myocardial [5,6] and skeletal muscles [7]. The metabolic maladaptation of mitochondria in CVD was observed with impaired oxidative phosphorylation (OXPHOS) and decreased ATP levels, increased apoptosis, deregulated autophagy, and severe oxidative stress [8,9]. Endothelial damage induces excessive ROS, which increases the endothelial permeability and monocytes adhesion to the vascular wall, subsequently promoting plague formation [10,11]. Proinflammation induced by mitochondrial dysfunction triggers plaque rupture, resulting in myocardial infarction and cerebrovascular accident (CVA) [12].

Despite the optimal pharmacological treatment, the progression of CVD often results in prognosis and low quality of life. Mitochondria’s role in CVD have been giving novel insights into finding therapeutic ways to fight the disease. However, it is challenging to activate mitochondrial biogenesis without compromising safety, to restore oxidative capacity in cardiomyocyte and vascular cells [2,13]. Therefore, mitochondria-targeted drug therapy remains elusive. Physical exercise is long known as a cardioprotective factor and reduces mortality. A recent cohort study with 30 years of follow-up revealed that long term physical activity was strongly associated with lower mortality for individuals who performed ≈150 to 300 min/week of vigorous physical activity, 300 to 600 min/week of moderate physical activity, or an equivalent combination of both [14]. Regular exercise reduces cardiovascular risks by down-regulating blood pressure, body weight, and LDL cholesterol, increasing HDL and insulin sensitivity, attenuating inflammation, and more recently, modifies mitochondrial dynamics and function [3]. 

Animal and pre-clinical studies have been suggesting exercise-induced effects on the mitochondrial life cycle, such as increasing PGC-1α, promoting fusion, enhanced oxygen consumption rate, and increased mitophagy. These benefits are yet to be elucidated in human study. Our previous studies demonstrated that moderate-intensity continuous and high-intensity interval exercise regimens improved the mitochondrial functions of platelets and lymphocytes in sedentary individuals [15,16,17,18] and in patients with CVD [19,20,21,22]. Nevertheless, additional information is needed to understand the exact mechanism of exercise on mitochondrial functionality in patients with CVD [23,24,25]. Moreover, the prescription of exercise, including type, intensity, duration, frequency, and progression, to ameliorate mitochondrial function, remained unclear.

In this regard, we aimed to clarify the effect of exercise training on mitochondrial function in people with CVD and to assemble the exercise protocols. We hypothesized that exercise promotes change on mitochondrial quantity, biogenesis, fusion/fission, and oxidative capacity, in different types of cells. The purpose of this systematic review and meta-analysis was to evaluate how exercise training modifies mitochondrial function in CVD, and thus to provide evidence-based guidance to clinicians in exercise prescription for patients with CVD.

## 2. Methods

This systematic review was conducted following the PRISMA (Preferred Reported Items for Systematic Review and Meta-analysis) guidelines [26].

### 2.1. Search Strategy

A literature search was undertaken on 30 June 2022, in PubMed and Scopus for articles written in the English language, adopting the search string presented in Table 1. Reference of all the searched articles have been screened for potential papers. The work was performed independently and synchronously by two of the authors (AY and YC).

### 2.2. Inclusion and Exclusion Criteria

The articles included in the analysis were required to fulfil the following inclusion criteria, in accordance with the PICO model:

Participants (P)-participants with cardiovascular disease, as defined by MeSH terms.

Intervention (I)-physical exercise as main intervention.

Comparator (C)-other intervention or control group with no intervention.

Outcomes (O)-mitochondrial outcomes, including morphology (size, shape, volume density, etc.), biogenesis (PGC-1α, Tfam, etc.), dynamics (fusion and fission phase), oxidative capacity (OXPHOS, MAPR, CS, etc.), antioxidant capacity (SOD, CAT, etc.), and quality (SIRT, COX, etc.).

Exclusion criteria are review, animal studies, non-experimental studies, studies involved subjects with hereditary disease, study with only one bout of exercise, articles written in languages other than English or have no available full text. In cases of disagreement between the two authors, a third author (JS) made a final decision on whether to include or exclude the article.

### 2.3. Data Extraction and Analysis

The articles searched were downloaded into the Endnote software (version X9) and duplicates were eliminated. Two authors (AY and YC) screened through the abstracts and read full text of the articles that met the inclusion criteria. Eligible articles were evaluated and the following information was extracted: (1) first author’s name; (2) year of article published; (3) type of participant disease; (4) study design; (5) sample size of the study; (6) age of the subjects; (7) exercise intervention: program, type of training, protocol duration, frequency, volume per session, intensity, control/healthy control group activity; (8) source of mitochondria; (9) mitochondria variables; and (10) post training change of mitochondrial variables.

### 2.4. Quality Assessment and Risk of Bias

The methodological quality of the studies was assessed by the two authors (AY and YC), according to the Tool for the Assessment of Study Quality and Reporting in Exercise (TESTEX) [27], which is designed specifically for use in exercise training studies. This tool consists of 15 items, including five items for study quality and 10 items for reporting. If the item is answered with “yes”, it is associated with a point. The Cochrane risk-of-bias tool for randomized trials (RoB2) [28] was used to assess the risk of bias of studies included in meta-analysis.

## 3. Results

### 3.1. Study Selection

**Figure 1:**
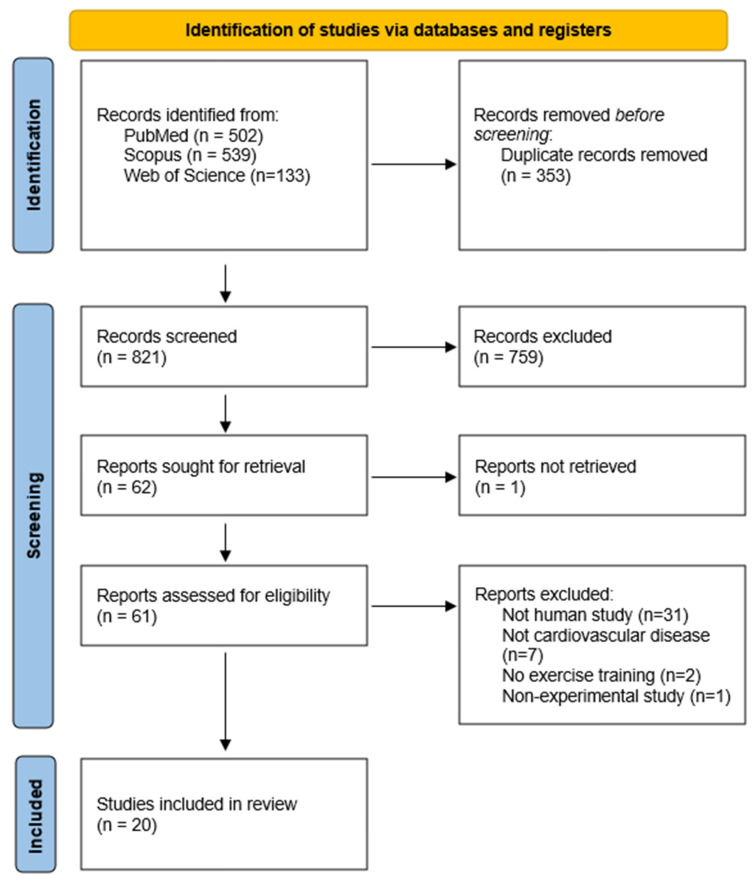
PRISMA flow diagram of the study. Review papers, animal studies, non-experimental studies, studies involving subjects with hereditary disease, studies with only one bout of exercise, and articles written in languages other than English, or have no available full text, were excluded.

The search in PubMed, Scopus and Web of Science identified 502, 539, and 133 articles, respectively. After duplication removal, 821 records were screened for titles and abstracts. Subsequently, 759 records were excluded, and the remaining 62 records were sought for full text. One paper could not be retrieved, resulting in 61 reports for a full reading. Finally, 41 articles were not eligible and thus 20 articles were included in the systematic review. Figure 1 shows the PRISMA flow diagram for the search process.

### 3.2. Study Characteristics

The methodological quality scores based on the TESTEX scale ranged from four to 13, with a mean of 8.85, of which 14 were above the average and six were below (Table 2).

The data of selected articles are presented in detail in Table 3, a total of 428 participants were included and 229 were in the training groups. Thirteen studies concerning heart failure (HF) (total subject *n* = 237) [22,29,30,31,32,33,34,35,36,37,38], four with peripheral artery disease (PAD) (total subject *n* = 112) [20,39,40,41,42], and two with CVA (total subject *n* = 30) [19], and each with coronary artery disease (total subject *n* = 12) [43], and hypertension (total subject *n* = 37) [44]. The sample size for the training group ranged between six and 24 individuals, with a mean age of 50 ± 12 to 76.5 ± 9 years. Thirteen studies were randomized controlled trial [19,20,22,29,32,33,38,39,40,41,42,43,45], including one crossover design [29]. Five of the seven quasi-experiments used healthy group for comparison [30,31,35,37,44] and the other two involved only one group pre-posttest [34,36]. The articles were published between the years 1993 and 2021.

**Table 2:** Quality assessment of the studies according to the TESTEX scale (*n* = 20).

Criteria	Eligibility Criteria Specified	Randomization Specified	Allocation Concealment	Groups Similar at Baseline	Blinding of all Assessors	Outcome Measures Assessed in 85% of Patients	Adverse Events Reported	Session Attendance Reported	Intent to Treat Analysis	Comparison Between Groups-Primary Outcome	Comparison between Groups-Secondary Outcome(s)	Point and Variability Measures	Activity Monitoring in Control Groups	Relative Exercise Intensity Remained Constant	Exercise Volume Characteristics and Energy Expenditure	Total
Chou 2019 [22]	Yes	Yes	No	Yes	Yes	Yes	No	Yes	Yes	Yes	Yes	Yes	No	Yes	Yes	12/15
Groennebaek 2019 [32]	Yes	Yes	No	Yes	No	Yes	Yes	Yes	No	Yes	Yes	Yes	No	Yes	Yes	11/15
Esposito 2018 [30]	No	No	No	Yes	No	Yes	No	Yes	No	Yes	Yes	Yes	No	Yes	Yes	8/15
Southern 2015 [35]	Yes	No	No	Yes	No	No	No	No	No	Yes	Yes	Yes	No	Yes	Yes	7/15
Toth 2012 [37]	No	No	No	Yes	No	Yes	No	No	No	Yes	Yes	Yes	No	Yes	Yes	7/15
Esposito 2011 [31]	No	No	No	Yes	No	Yes	No	Yes	No	Yes	Yes	Yes	No	Yes	No	7/15
Williams 2007 [38]	Yes	Yes	No	Yes	No	Yes	No	Yes	No	Yes	Yes	Yes	No	Yes	No	9/15
Wisløff 2007 [42]	Yes	Yes	No	Yes	No	Yes	Yes	No	No	Yes	Yes	Yes	Yes	Yes	Yes	11/15
Santoro 2002 [34]	Yes	No	No	No	No	Yes	No	No	No	No	No	Yes	No	Yes	Yes	5/15
Hambrecht 1997 [45]	No	Yes	No	Yes	Yes	No	No	No	No	Yes	Yes	Yes	No	Yes	Yes	8/15
Hambrecht 1995 [33]	No	Yes	No	Yes	Yes	No	No	No	No	Yes	Yes	Yes	No	Yes	Yes	8/15
Stratton 1994 [36]	No	No	No	No	No	Yes	Yes	Yes	No	No	No	Yes	No	No	No	4/15
Adamopoulos 1993 [29]	No	Yes	No	Yes	Yes	Yes	No	No	Yes	Yes	Yes	Yes	No	Yes	Yes	10/15
Lin 2021 [20]	Yes	Yes	No	Yes	Yes	Yes	Yes	Yes	Yes	Yes	Yes	Yes	No	Yes	Yes	13/15
Murrow 2019 [40]	Yes	Yes	No	Yes	No	No	No	Yes	No	Yes	Yes	Yes	No	Yes	Yes	9/15
van Schaardenburgh 2017 [41]	Yes	Yes	No	Yes	Yes	Yes	Yes	Yes	Yes	Yes	Yes	Yes	No	Yes	Yes	13/15
Hiatt 1996 [39]	No	Yes	No	Yes	No	No	Yes	No	No	Yes	Yes	Yes	No	Yes	Yes	8/15
Hsu 2019 [19]	Yes	Yes	No	Yes	Yes	No	No	Yes	Yes	Yes	Yes	Yes	No	Yes	Yes	11/15
Zoll 2006 [43]	No	Yes	No	Yes	No	No	No	No	Yes	No	No	Yes	No	Yes	Yes	6/15
Fiorenza 2019 [44]	Yes	No	No	Yes	No	Yes	No	Yes	Yes	Yes	Yes	Yes	No	Yes	Yes	10/15

### 3.3. Physical Exercise Programs

Eleven studies employed aerobic exercise using a static bike, treadmill, or functional movements, of which three used high intensity interval training (HIIT) [22,42,44]. Eight studies involved resistance exercise, of which only three worked on multiple muscle groups [34,37,38], the others were knee or hand movements. One study explored blood flow restricted knee extension exercise with low intensity [32]. Two studies had group training in addition to cycling regimen [33,45].

The exercise intensity in aerobic training was tailored to participants’ characteristics, based on peak oxygen uptake (% VO_2peak_, 60%) [19,22,43], the percentage of peak heart rate (%HR_peak_, 70~95%) [33,45], the percentage of maximum heart rate (%HR_max_, 70~80%) [29,42], ventilation threshold (VT) [20], and pain threshold [39,40,41]. The training duration ranged from four to 24 weeks, with a frequency of twice a week to daily. Time per session ranged from 10 to 60 min.

Resistance training was performed with free weights or machines. The exercise intensity was tailored to participants’ characteristics, based on Repetition Maximum (RM, ranged 30~80%) [32,34,37], maximum voluntary isometric contraction (MVIC, 15%) [35], or maximum work rate (WR_max_, 50~100%) [30,44]. The training duration ranged from 6 to 18 weeks, with a frequency of thrice a week to daily. Each session took 10 to 100 min or 2–3 sets of 8–10 repetitions.

In terms of the disease cohorts, five of the 13 HF studies adopted aerobic exercise and the others were resistance exercise. Three studies compared the training effect between patients and healthy control [30,35,37]. Three studies gave advice or no intervention to the control group [22,32], three continued with usual care, while the others had no control group. Concerning PAD, three of the four studies used functional activities such as walking [39,40,41], calf raises [41], or lower limb movement [39]. Two studies recruited control group with the usual care/general rehab program [19,20]. Aerobic exercise was implemented in studies for CVA, coronary artery disease, and hypertension.

More details of the exercise characteristics of each included study are presented in Table 3.

### 3.4. Mitochondrial Outcomes

Table 4 shows the results of mitochondrial modification upon exercise training. The selected studies performed muscle biopsy, spectroscopy, or venous blood test to evaluate the mitochondria function of skeletal muscle cells or platelets. The majority of the studies performed skeletal muscle biopsy on either vastus lateralis [30,31,32,33,34,37,38,42,43,44,45] or gastrocnemius [39,41]. Four studies used spectroscopy to examine skeletal muscle mitochondrial function from the forearm [35,36] and lower leg [29,40]. Chou et al. [22], Lin et al. [20], and Hsu et al. [19] had withdrawn venous blood to analyze platelet mitochondrial bioenergetics in patients with HF, CVA, and PAD, respectively.

We assessed the mitochondrial outcomes in terms of morphology, biogenesis, dynamics, oxidative capacity, antioxidant capacity, and quality.

#### 3.4.1. Mitochondrial Morphology

Two studies on patients with HF reported controversial findings on the effect of resistance training in mitochondrial phenotypes. Toth et al. [37] showed no change to the muscle mitochondrial size after 18 weeks of moderate-intensity systemic resistance exercise, whereas Santoro et al. [34] reported an increase of 23.4% in size with unaltered shape with similar protocol but higher intensity.

#### 3.4.2. Mitochondrial Biogenesis and Dynamics

Two studies [30,31] investigated the effect of resistance training on the mitochondrial volume density in patients with HF and reported significant gain in the mitochondrial volume density. Another two studies [33,45], examined the effect of aerobic exercise, and also reported significant increase in the mitochondrial volume density. In detail, there were increment in TVVM (19%), the surface density of the mitochondrial inner border membrane—SVM_IMB_ (92%), the surface density of mitochondrial cristae—SVMC (43%), and the surface density of cytochrome c oxidase-positive mitochondria—SVMO_COX+_ (between 27 and 41%).

In studies that evaluated the protein and enzyme activities contributing to mitochondrial biogenesis, there was no agreement from the selected studies. Two studies [37,43] showed no change in PGC-1α, whereas Wisløff et al. [42] reported a significant increment after 12-week AIT. One study [37] reported that Tfam improved significantly after training, while the other [43] showed no change. Fiorenza et al. [44] reported no change in ERRα after training. The one study [44] that investigated the fusion and fission phase reported that MFN1 and OPA1 were down-regulated by HIIT. MFN2 was up-regulated by HIIT but DRP1 had no change.

#### 3.4.3. Mitochondrial Oxidative Capacity

**Figure 2:**
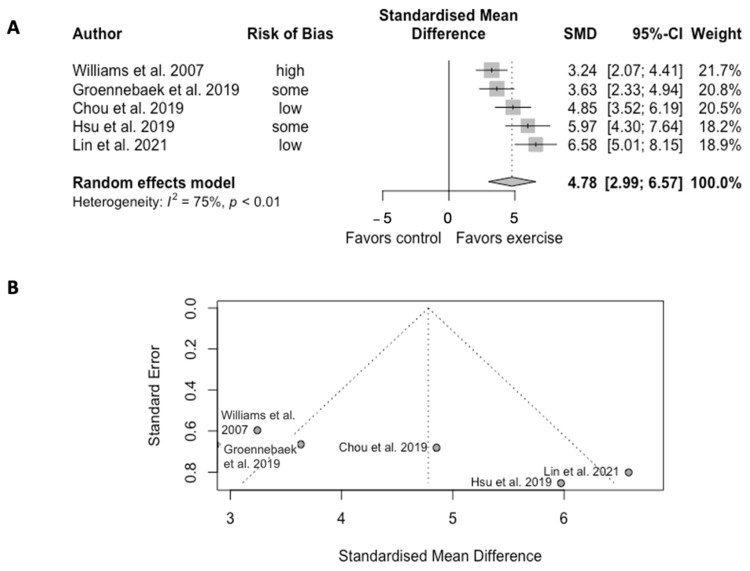
(**A**) Forest plot of effect sizes and 95% confidence intervals representing oxidative capacity, based on the random effects meta-analysis results. (**B**) Funnel plots of publication bias. Abbreviation: SMD, standardized mean difference. Williams et al. [38], Groennebaek et al. [32], Chou et al. [22], Hsu et al. [19], and Lin et al. [20].

Exercise training significantly increased the mitochondrial oxidative capacity in five trials (SMD = 4.78, CI = 2.99 to 6.57, *p* < 0.01) (Figure 2). However, the analysis showed high heterogeneity among studies (Q = 16.10, df = 4, *p* = 0.003, I^2^ = 75%).

Eleven papers reported mitochondrial oxidative capacity, of which two reported resting muscle oxygen consumption (mVO_2_), five studies examined electron transfer system (ETS) or OXPHOS [19,20,22,32,41], three studies reported the mitochondrial ATP production rate (MAPR) [29,36,38], two studies assessed phosphocreatine (PCr) measures [29,33], and six studies measured citrate synthase activity [32,37,38,39,41,44].

Chou et al. [22] reported a 12-week of HIIT cycling regimen significantly increased the maximal and reserve platelet OCR capacities, and enhanced the Complex I- and II-mediated OCRs from ETS activity in patients with HF. Groennebaek et al. [32] studied blood flow restricted knee extension exercise and reported a 6-week training increaseof 23% of the state 3 respiration supported by complex I and II in patients with HF. Lin et al. [20] reported 12-week cycling at ventilation threshold significantly enhanced succinate-involved OXPHOS level, maximal OXPHOS and ETS in platelet in patients with PAD. On the other hand, van Schaardenburgh et al. [41] reported that the 8-week home-based walking/calf raise exercise improved neither the capacity of OXPHOS nor ETC in patients with PAD, but the calf raise training group had significant improvement in the CS activity in platelet. Hsu et al. [19] studied platelet mitochondrial bioenergenetics in CVA patients and reported that the 4-week cycling plus general rehab significantly enhanced OXPHOS and ETS by activating the FADH2 (Complex II)-dependent pathway. Southern et al. [35] and Murrow et al. [40] showed controversial results regarding the effects of exercise training on mVO_2_. One showed no change in patients with HF, while the other showed significant improvement in subjects with PAD after training.

Three studies, involved patients with HF, reported the effects of exercise training in MAPR. Adamopoulos et al. [29] did a RCT studying the effect of an 8-week cycling program on the oxidative capacity of calf muscle cells in patients with HF; the study reported that reduced PCr recovery half-time with improved MAPR after training were observed through spectroscopy. Meanwhile, Straton et al. [36] reported similar findings of increased PCr resynthesis rate with improved MAPR in forearm muscle cells, also observed through spectroscopy, after 4-week daily hand exercise using hand-held dynamometer. A RCT performed by Williams et al. [38] confirmed the improvement in MAPR, through muscle biopsy, after 3 months of circuit resistance exercise. This study also reported an increase in CS activity.

There are controversial findings regarding the enzymatic activity of CS. Four studies reported a significant increase in CS activity followed by their training protocol [32,38,41,44], whereas the other training protocol of three studies reported no change or reduction in CS activity [39,41]. The 18-week resistance training protocol involving patients with HF, performed by Toth et al. [37], showed no effect in CS activity. Hiatt et al. [39] reported reduced and no change in CS activity after 12 weeks of lower limb resistance exercise and walking, respectively, in patients with PAD. Similarly, the 8-week home-based walking protocol by van Schaardenburgh et al. [41] also showed no effect in CS activity.

#### 3.4.4. Mitochondrial Antioxidant Capacity and Quality

Only one study from the selected studies investigated the effects of training on mitochondrial antioxidant capacity. Fiorenza et al. [44] reported upregulated SOD2 but decreased SOD1, along with augmented CAT and NOX. Divergent responses were seen in the markers of mitochondrial antioxidant protection. Two studies evaluated the mitochondrial quality. Zoll et al. [43] reported no change in COX after aerobic exercise in patients with coronary artery disease. Fiorenza et al. [44] showed a lack of change in COX-IV and SIRT3 abundance after HIIT cycling in people with hypertension. No alteration was observed in the mitochondrial quality as explained by the two studies.

## 4. Discussion

**Figure 3:**
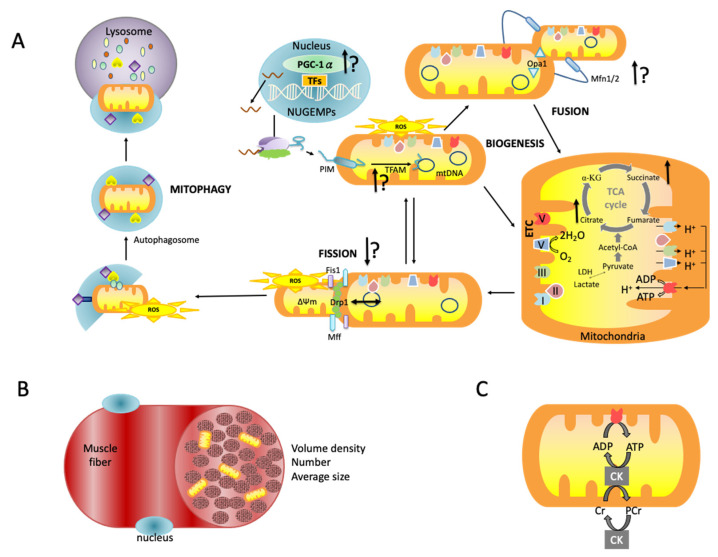
Illustrative diagram shows: (**A**) the mitochondrial life cycle with suggesting effect of exercise training; (**B**) the mitochondrial quantity in muscle cells; and (**C**) ATP synthesis. ↑ indicates majority evidence of exercise-induced benefits; ↑? indicates some evidence of exercise-induced benefits but further evidence needed; ↔ indicates majority evidence of exercise-induced benefits.

This is the first systematic review and meta-analysis to investigate the effects of exercise training on mitochondrial function in patients with CVD. Notably, the meta-analysis indicated high effect size on the training group in improving mitochondrial oxidative capacity in individuals with HF, CVA, and PAD. The exercise intensity, rather than the type, is the key player in this modifying effect. Figure 3 shows the suggesting effect of exercise training in the pathway of mitochondrial life cycle.

### 4.1. Study Quality

The studies in this systematic review included 13 RCTs, five quasi-experiments, and two one-group pre-posttest. According to the TESTEX scored, RCTs ranged from six to 13 points, quasi-experiments ranged from five to 10 points, and one-group pre-posttest scored four and five points. Reasonably, the articles with lower scores were published in earlier years, and vice versa. The quasi-experiments conducted exercise on CVD patients and age-matched non-patients. The findings, although inadequate, allowed a glance of how CVD patients reacted to exercise as compared to healthy individuals. Under the similar exercise regime, CVD patients demonstrated more improvement in mitochondrial functionality than healthy individuals.

Only oxidative capacity was eligible to be performed on meta-analysis. The pooled articles showed the large effect size of exercise training on the oxidative capacity but with significant heterogeneity, which was further explained by the funnel plot. Possible systematic heterogeneity presented due to the cells examined, of which three studies reported platelet mitochondria, while the other two reported skeletal muscle mitochondria. Nevertheless, mitochondria in both cells demonstrated enhanced succinate-phase in OXPHOS after exercise training.

### 4.2. Exercise and Mitochondrial Oxidative Capacity

Three studies [19,20,22] included in the review demonstrated aerobic training enhanced platelet mitochondrial OXPHOS and ETS capacities through accelerated complex II activity in patients with PAD, CVA, and HF. Exercise-induced elevation in complex II activity may eliminate succinate, further decreasing ROS production from platelet mitochondria, eventually depressing systemic oxidative stress and proinflammatory status in patients with CVD. This phenomena of enhanced succinate-phase in OXPHOS also presented in the study involved in skeletal muscle [32]. Earlier studies on exercise intervention predominantly focused on mitochondrial functionality in skeletal muscles [46]. However, peripheral blood cells, circulating markers in the cardiovascular system, serve a better role in explaining the change of the system. Platelet is one of these markers, given the closely-knit relationship with cardiovascular presentation.

Furthermore, blood-flow restricted resisted exercise (BFRRE) at low intensity increased coupled mitochondrial respiration in skeletal muscle against oxidative stress by increasing Complex II activity in HF patients [32]. BFRRE increased RNA synthesis rate in skeletal muscle instead of the protein synthesis rate possibly attributed to the reverse of impaired anabolic sensitivity in patients with CHF [47]. This is in accordance with previous in vitro study that reported exercise with oxygen restriction attenuated mitochondrial ROS emission rates and the fraction of electron leak to ROS compared to room air. This is similar to our previous study reported enhanced removal of ROS followed the exercising under hypoxic condition [2].

### 4.3. Exercise and Mitochondrial Biogenesis

Mitochondrial production is stimulated by the PGC-1α-NRF1-TFAM pathway. PGC-1α is the first stimulator of mitochondrial biogenesis. NRF1 is an intermediate transcription factor, which stimulates the synthesis of TFAM, which is a final effector activating the duplication of mitochondrial DNA molecules. PGC-1α is understood as an important mediator to the known positive outcomes of physical exercise on skeletal muscle physiology [48], the dysregulation causes energetic impairment in HF [49].

High-intensity aerobic interval training at 90% to 95% HR_peak_ with interval pauses walking at 50% to 70% HR_peak_ improved PGC-1α, whereas moderate-intensity continuous aerobic exercise at 70% HR_peak_ did not promote change in PGC-1α in HF patients [42]. Previous studies showed that the activation of PGC-1α upon an acute bout of endurance exercise, but not intense, activated signaling events and increased ROS [50,51]. Animal studies showed that PGC-1α deletion reduces mitochondrial content and respiration capacity, and endurance performance could reverse the impact [52,53]. The modification on the mitochondrial biogenesis seems to be intensity-based in the aerobic exercise, whereas findings reported effect of resistance exercise on PGC-1α is contradicted.

Transcription factor A (TFAM) expression was enhanced by resistance exercise on major muscles groups at high intensity (80% 1RM) [37] in both HF patients and healthy elderly, but no changes were noted in NRF1. TFAM has linked to muscle atrophy, and animal study suggested a combination of exercise and TFAM leads to an interactive effect in targeting mitochondrial function to prevent skeletal muscle atrophy [54].

### 4.4. Exercise and Mitochondrial Morphology, Antioxidant Capacity and Quality

Moderate intensity exercises improved mitochondrial volume and density in patients with HF [30,31,33,45]. Mitochondrial volume and density are associated with capillarity [30], indicating skeletal muscle metabolic adaptation and the vasculature. HIIT cycling consisted of intervals of aerobic and anaerobic stimulation, which could promote cardiac remodeling in HF patients [55]. The benefits of exercise on skeletal muscle in people with CVD have long been discussed and was understood to be able to enhance mitochondrial function, and thus restoration and the improvement of vasculature. Skeletal muscles involved high metabolic activities to support the intensive myofiber contractility and produced myokines, which play a key role in energy homeostasis [56]. Myokines released from skeletal muscle, acting as circulating hormones, preserve or improve cardiovascular function [57].

Cardiovascular risks and disorders, including HF, PAD, CVA, coronary artery disease, and hypertension, present with mild to severe symptoms. Prolonged hypertensive condition often leads to pathologic hypertrophy of the ventricular walls, eventually compromising myocardial function and resulted in HF [57]. Hypertensive individuals that have undergone 6 weeks of HIIT cycling had enhanced endothelial NOS and VEGF expressions, as well as mitochondrial biogenesis and autophagy [44]. At the same time, the HIIT created varied responses in mitochondrial fusion and the antioxidant capacity had no effect on markers of mitochondrial fission, but increased the markers of mitochondria-mediated apoptosis and oxidative damage in skeletal muscle from the individuals with hypertension. The study showed a correlation between oxidative stress and elevated blood pressure but did not analyze the relationship between physical performance, endothelial markers, and mitochondrial markers. In animal studies, exercise training reduced ROS formation and restored OXPHOS by regulating SIRT1, which improved endothelial function and attenuates aortic stiffening [24,58].

### 4.5. Study Limitation

This review has a few limitations that preclude drawing a whole picture on the effect of exercise training in people with CVD. First, no cardiac muscles were examined in the selected studies, largely due to the low possibility to retrieve the cardiomyocytes in human at pre and post exercise training. However, we proposed and proved that peripheral blood cells, such as platelets, could mirror the myocardial bioenergetic. Second, several selected papers had moderate and below quality due to non-RCT design. As shown in the meta-analysis, oxidative capacity had stronger evidence but remained highly heterogeneous. Future investigation involving exercise intervention should consider a randomized controlled study design. Third, publication dates were ranged across three decades. Measuring methods and tools could have evolved throughout the years, causing bias in the interpretation. A more focus measurement on the role of exercise examining mitochondrial modification warrants further investigation.

Despite these limitations, this meta-analysis study has significant implications to clinical application, providing a glance of exercise protocols used for CVD to modify mitochondrial function in the published clinical study. Moreover, it is noteworthy to observe how exercise training modifies the mitochondrial dysfunction in humans since most of the theories remained in vitro/animal studies. Physical exercise comes in various forms, therefore knowing the principle of exercise provides a more optimized exercise prescription for CVD patients through the mitochondrial-targeted strategy.

## 5. Conclusions

Exercise training improves the mitochondrial oxidative capacity of both skeletal muscles and platelets in patients with CVD. However, exercise protocols and outcomes were diverse with controversy regarding the mitochondrial functions of other cells. Therefore, further research is required to provide more evidence of the effectiveness of exercise training on myocardial mitochondrial function.

## Figures and Tables

**Table 1:** Search strategy used in literature search.

Databases	Search String
PubMed	((mitochondria*[Title/Abstract]) AND ((exercise[Title/Abstract]) OR (aerobic capacity[Title/Abstract])) AND (cardiovascular disease[MeSH Terms])) NOT (review[Publication Type])
Scopus	(TITLE-ABS-KEY(mitochondria*) AND TITLE-ABS-KEY(exercise) AND TITLE-ABS-KEY ( "cardiovascular disease" OR "heart failure" OR "myocardiac infarct" OR stroke OR "peripheral artery disease")) AND (LIMIT-TO(DOCTYPE, "ar" )) AND (LIMIT-TO (LANGUAGE, "English" ))
Web of Science	mitochondria (All Fields) and exercise (All Fields) and cardiovascular disease (All Fields)

**Table 3:** Summary of included studies (*n* = 20).

Authors	Disease	Study Design	Subject	Age	Exercise Program	Exercise Intervention					
						Type of Training	Protocol Duration	Frequency	Volume per Session	Intensity	Control/Healthy Group Activity
Chou et al., 2019 [22]	Heart failure	RCT	N: 34 TG: 17 CG: 17	TG: 60.9 ± 0.5 CG: 59.7 ± 5.3	Aerobic	HIIT cycling	12 weeks	5 days/week	30 min	Five 3-min intervals at 40%, 80% VO_2peak_	CG: General health consultation
Groennebaek et al., 2019 [32]	Heart failure	RCT	N: 36 TG: 12 RICG: 12 CG: 12	TG: 66 ± 7 RICG: 62 ± 9 CG: 63 ± 10	Resistance	Blood flow restricted knee extension	6 weeks	3 days/week	4 sets	30% of 1RM	CG: no intervention RICG: once daily, 4 cycles of 5 min upper arm ischemia followed by 5 min of reperfusion
Esposito et al., 2018 [30]	Heart failure	Quasi-experiment	N: 12 TG: 6 HG: 6	TG: 54 ± 14 HG: 51 ± 8	Resistance	Knee extension	8 weeks	3 days/week	100 min	50% WR_max_	HG: one-time assessment
Southern et al., 2015 [35]	Heart failure	Quasi-experiment	N: 12 TG: 7 HG: 5	TG: 66 ± 4.1 HG: 61 ± 5.5	Resistance	Non-dominant wrist flexor training with hand weight set	4 weeks	4 days/week	30 min	15% MVIC	HG: same training regimen
Toth et al., 2012 [37]	Heart failure	Quasi-experiment	N: 23 TG: 10 HG: 13	TG: 71.8 ± 3.4 HG: 71.7 ± 1.7	Resistance	Leg extension, leg press, leg curls, shoulder press, bench press, bicep curls, and lat pull-downs	18 weeks	3 days/week	Week 1: 50% of 1RM, 1 set 10 reps Week 2: 60% of 1RM, 2 sets 8 reps Week 3: 70% of 1RM, 3 sets 8 reps Week 4~18: 80% of 1RM, 3 sets 8 rep		HG: same training regimen
Esposito et al., 2011 [31]	Heart failure	Quasi-experiment	N: 12 TG: 6 HG: 6	TG: 54 ± 14 HG: 51 ± 8	Resistance	Knee extension	8 weeks	3 days/week	100 min	Not mentioned	HG: one-time assessment
Williams et al., 2007 [38]	Heart failure	RCT	N: 13 TG: 7 CG: 6	TG: 67 ± 9 CG: 74 ± 4	Resistance	Circuit training (Leg cycle, elbow extension/flexion, stair climbing, arm cycling, knee extension, and shoulder press)	11 weeks	3 days/week	0.5~2 min for each	Moderate	Usual care
Wisløff et al., 2007 [42]	Heart failure	RCT	N: 27 MCTG: 9 AITG: 9 CG: 9	MCTG: 74.4 ± 12 AITG: 76.5 ± 9 CG: 75.5 ± 13	Aerobic	MCT or AIT Treadmill	12 weeks	3 days/week	MCTG: 47 min AITG: 38 min	MCTG: 70% HR_peak_ AITG: 95% HR_peak_	CG: Standard advice regarding physical activity
Santoro et al., 2002 [34]	Heart failure	One group pre-posttest	N: 6 TG: 6	TG: 73 (67~82)	Resistance	Leg press, knee curl, chest press, upper back machine, knee extension	16 weeks	3 days/week	2 sets of 8 repetitions	40~60% 1RM	No CG/HG
Hambrecht et al., 1997 [45]	Heart failure	RCT	N: 18 TG: 9 CG: 9	TG: 50 ± 12 CG: 52 ± 8	Aerobic	Cycling + group training (walking, calisthenics and ball games)	24 weeks	Week 1–3: Cycling 6 times/day, 10 min each (inpatient) Week 4–24: Cycling 2 times/day, total 40 min (home); and group training 2 days/week		70% HR_peak_	Usual care
Hambrecht et al., 1995 [33]	Heart failure	RCT	N: 22 TG: 12 CG: 10	TG: 50 ± 12 CG: 52 ± 8	Aerobic	Cycling + group training (walking, calisthenics and ball games)	24 weeks	Week 1–3: Cycling 6 times/day, 10 min each (inpatient) Week 4–24: Cycling 2 times/day, total 40 min (home); and group training 2 days/week		70% HR_peak_	Usual care
Stratton et al., 1994 [36]	Heart failure	One group pre-posttest	N: 10 TG: 10	TG: 62 ± 11	Resistance	Hand-held dynamometer	4 weeks	7 days/week	2–3 sets of 10 reps of 10 s hold + 5 min of repeatedly squeezing	Not mentioned	No CG/HG
Adamopoulos et al., 1993 [29]	Heart failure	RCT crossover with HG comparison	N: 39 TG: 12 CG: 12 HG: 15	TG: 62.4 ± 2.6 HG: 55.2 ± 2.8	Aerobic	Cycling	8 weeks	5 days/week	20 min	70~80% HR_max_	CG: avoidance of exercise HG: one-time assessment
Lin et al., 2021 [20]	Peripheral artery disease	RCT	N: 40 TG: 20 CG: 20	TG: 71.1 ± 1.5 CG: 70.5 ± 1.9	Aerobic	Cycling + general rehab	12 weeks	3 days/week	30 min	At VT	CG: General rehab
Murrow et al., 2019 [40]	Peripheral artery disease	RCT	N: 18 NTG: 8 WTG: 10	NTG: 72.0 ± 9.7 WTG: 71.6 ± 8.8	Aerobic	Walking on treadmill	12 weeks	3 days/week	30 min	NTG: Tissue saturation index reduced >15% WTG: claudication pain rating ≧6	No CG/HG
van Schaardenburgh et al., 2017 [41]	Peripheral artery disease	RCT	N: 27 TTG: 13 CRTG: 14	TTG: 70 ± 8.2 CRTG: 66 ± 9.3	Aerobic vs. Resistance	Walking on treadmill vs. calf raises	8 weeks	TTG: 3 days/week CRTG: 3 times/day	TTG: 30 min CRTG: not specified	TTG: near pain threshold CRTG: 5 more calf raise after pain felt	No CG/HG
Hiatt et al., 1996 [39]	Peripheral artery disease	RCT	N: 26 TTG: 10 STG: 8 CG: 8	TTG: 67 ± 7 STG: 67 ± 6 CG: 67 ± 5	Aerobic vs. Resistance	Walking on treadmill vs. lower limb exercise with cuff weight	12 weeks	3 days/week	TTG: 60 min STG: 3 sets of 6 reps	TTG: moderate claudication pain STG: not mentioned	Usual care
Hsu et al., 2019 [19]	Stroke	RCT	N: 30 TG: 15 CG: 15	TG: 55.7 ± 3.0 CG: 57.8 ± 3.9	Aerobic	Cycling + general rehab	4 weeks	5 days/week	30 min	60% VO_2peak_	CG: General rehab
Zoll et al., 2006 [43]	Coronary artery disease	RCT	N: 12 CETG: 6 EETG: 6	Not mentioned	Aerobic	Cycling	8 weeks	3 days/week	30 min	60% VO_2peak_	No CG/HG
Fiorenza et al., 2019 [44]	Hypertension	Quasi-experiment	N: 37 TG: 24 HG: 13	TG: 58.4 ± 2.5 HG: 60.8 ± 1.5	Aerobic	HIIT Cycling	6 weeks	2~3 days/week	2~3 sets of 5 min bout	30~100% WR_max_	HG: same training regimen

**Table 4:** Results on mitochondrial modification.

Author Year	Source of Mitochondria	Variables	Training	Healthy	Control
Chou et al., 2019 [22]	Blood sample (platelet)	OXPHOS (pmol/s/10^8^ cells)	↑ *	-	↔
		ETS (pmol/s/10^8^)	↑ *	-	↔
Groennebaek et al., 2019 [32]	Muscle biopsy (vastus lateralis)	OXPHOS (pmol/s/mg)	↑ **	-	CG ↔ RIC ↔
		CS (μmol/min/g)	↔	-	CG ↔ RIC ↔
Esposito et al., 2018 [30]	Muscle biopsy (vastus lateralis)	VVM (%)	↑ *	-	-
Southern et al., 2015 [35]	Spectroscopy (wrist flexors)	Rate of recovery of mVO_2_ (min^−1^)	↔	↑ *	-
Toth et al., 2012 [37]	Muscle biopsy (vastus lateralis)	Fractional density (%)	↔	↔	-
		Number (μm^2^/area)	↔	↔	-
		Average size (μm^2^)	↔	↔	-
		PGC-1α (% of control)	↔	↔	-
		PGC-1β (% of control)	↔	↔	-
		NRF-1 (% of control)	↔	↔	-
		TFAM (% of control)	↑ **	↑ **	-
		COX-1 (% of control)	↔	↓ **	-
		COX-5b (% of control)	↔	↔	-
		COX (μmol/min/mg)	↔	↔	-
		CS (μmol/min/mg)	↔	↔	-
Esposito et al., 2011 [31]	Muscle biopsy (vastus lateralis)	VVM (%)	↑ *	-	-
Williams et al., 2007 [38]	Muscle biopsy (vastus lateralis)	MAPR (mmol ATP/min/kg)	↑ *	-	↓ *
		CS (mmol/min/kg)	↑ **	-	↔
		HAD (mmol/min/kg)	↔	-	↔
		PFK (mmol/min/kg)	↔	-	↔
		LDH (mmol/min/kg)	↔	-	↔
Wisløff et al., 2007 [42]	Muscle biopsy (vastus lateralis)	PGC-1α (arb. unit)	MCTG ↔ AITG ↑ **	-	↔
Santoro et al., 2002 [34]	Muscle biopsy (vastus lateralis)	Area (μ^2^)	↑ *	-	-
		Elongation	↔	-	-
Hambrecht et al., 1997 [45]	Muscle biopsy (vastus lateralis)	SVMO_cox+_ (m^2^/cm^3^)	↑ **	-	↔
		SVMO_cox-_ (m^2^/cm^3^)	↔	-	↔
		SVM_IBM_ (m^2^/cm^3^)	↑ **	-	↔
		SVMC (m^2^/cm^3^)	↑ **	-	↔
		VVM (%)	↑ **	-	↔
		N_cox+_ (*n*)	↔	-	↔
		N_cox-_ (*n*)	↔	-	↔
		SVMO_cox+_/N_SVMOcox+_ (m^2^/cm^3^)	↔	-	↔
Hambrecht et al., 1995 [33]	Muscle biopsy (vastus lateralis)	VVM_cox+_ (%)	↑ **	-	↔
		VVM_cox-_ (%)	↔	-	↔
		VVM (%)	↑ *	-	↔
Stratton et al., 1994 [36]	Spectroscopy (forearm muscle)	PCr resynthesis rate (mM/min)	↑ **	-	-
		MAPR (mM/min)	↑ *	-	-
Adamopoulos et al., 1993 [29]	Spectroscopy (calf muscle)	PCr recovery time (min)	↓ *	-	-
		PCr resynthesis rate (mmol/liter/min)	↑ *	-	-
		MAPR (mmol/liter/min)	↑ **	-	-
Lin et al., 2021 [20]	Blood sample (platelet)	ETS (pmol/sec/10^8^ cells)	↑ *	-	↔
		OXPHOS (pmol/sec/10^8^ cells)	↑ *	-	↔
Murrow et al., 2019 [40]	Spectroscopy (gastrocnemius)	Rate of recovery of mVO_2_ (rate constant)	NTG ↑ * WTG ↑ *	-	-
van Schaardenburgh et al., 2017 [41]	Muscle biopsy (gastrocnemius)	OXPHOS (pmol/mg/sec)	TTG ↔ CRTG ↔	-	-
		ETS (pmol/mg/sec)	TTG ↔ CRTG ↔	-	-
		CS (μmol/min/mg)	TTG ↔ CRTG ↑ *	-	-
Hiatt et al., 1996 [39]	Muscle biopsy (gastrocnemius)	LDH (mmol/min/g)	TTG ↔ STG ↔	-	TTG ↔ STG ↔
		CS (μmol/min/g)	TTG ↔ STG ↓*	-	TTG ↔ STG ↔
		PFK (mmol/min/g)	TTG ↑ * STG ↔	-	TTG ↔ STG ↔
		Carnitine (nmol/g)	TTG ↔ STG ↔	-	TTG ↔ STG ↔
Hsu et al., 2019 [19]	Blood sample (platelet)	OXPHOS (pmol/sec/10^8^ cells)	↑ *	-	↔
		ETS (pmol/sec/10^8^ cells)	↑ *	-	↔
Zoll et al., 2006 [43]	Muscle biopsy (vastus lateralis)	VVM (%)	CETG ↔ EETG ↓ *	-	-
		PGC-1α (mRNA level)	CETG ↔ EETG ↔	-	-
		TFAM (mRNA level)	CETG ↔ EETG ↔	-	-
		COX-1 (mRNA level)	CETG ↔ EETG ↔	-	-
		COX-4 (mRNA level)	CETG ↔ EETG ↓ *	-	-
Fiorenza et al., 2019 [44]	Muscle biopsy (vastus lateralis)	HAD (μmol/min/g)	↑ **	↔	-
		CS activity (μmol/min/g)	↑ ***	↔	-
		CS content (arb. unit)	↑ **	↔	
		COX IV (arb. unit)	↑ **	↔	
		ERRα(arb. unit)	↔	↔	-
		mitofusin 1 (arb. unit)	↓ **	↔	-
		mitofusin 2 (arb. unit)	↑ **	↔	-
		OPA1 (arb. unit)	↓ **	↔	
		Drp1 (arb. unit)	↔	↔	-
		LC3-I (arb. unit)	↔	↓ *	-
		LC3-II (arb. unit)	↔	↑ *	-
		LC3-II/LC3-I ratio (arb. unit)	↔	↑ **	-
		p62 (arb. unit)	↑ ***	↑ *	-
		BAX (arb. unit)	↑ ***	↑ *	-
		Bcl-2 (arb. unit)	↓ **	↔	-
		BAX/Bcl-2 ratio (arb. unit)	↑ ***	↑ ***	-
		SOD1 (arb. unit)	↓ **	↔	-
		SOD2 (arb. unit)	↑ *	↑ *	-
		GPX1 (arb. unit)	↔	↑ *	-
		Catalase (arb. unit)	↑ *	↔	-
		NOX (arb. unit)	↑ *	↑ *	-
		Sirtuin 3 (arb. unit)	↔	↔	-
		UCP3 (arb. unit)	↔	↔	
		HSP70 (arb. unit)	↓ **	↔	

Remarks: ETS: electron transport system; OCR: oxygen consumption rate; BHI: bioenergetic health index; GM: state 2 respiration; GM3: state 3 respiration supported by complex I; GMS3: state 3 respiration supported by complex I and II; 4o: state 4 respiration; RCR: respiratory control ratio CS: citrate synthase; VVM: mitochondrial volume density; mVO2: resting muscle oxygen consumption; MAPR: mitochondrial ATP production rate; HAD: β-hydroxyacyl coenzyme A dehydrogenase; PFK: phosphofructokinase; LDH: lactate dehydrogenase; CG: control group; RIC: remote ischemic conditioning; NTG: NIRS guided training group; WTG: walking training group. * Significantly different from baseline, *p* ≤ 0.05; ** Significantly different from baseline, *p* ≤ 0.01; *** Significantly different from baseline, *p* ≤ 0.001; ↑ increase observed, ↓ decrease observed, ↔ no change observed.
